# Mother to Mother: Mothers’ Social Comparison-Based Emotions on Social Networking Sites

**DOI:** 10.5964/ejop.v16i4.2159

**Published:** 2020-11-27

**Authors:** Leman Pinar Tosun, Ahu Öztürk, Gamze Özdemir

**Affiliations:** a Psychology Department, Bursa Uludag University, Bursa, Turkey; b Psychology Department, Çanakkale 18 Mart University, Çanakkale, Turkey; University of Liverpool, Liverpool, United Kingdom

**Keywords:** motherhood, social network sites, social comparison theory, social comparison-based emotions, assimilation vs. contrast

## Abstract

Social networking sites (SNSs) are platforms where people make social comparisons very frequently, and because of those comparisons, they have the potential for evoking a wide range of emotions. According to typology of social comparison-based emotions, the emotions felt after social comparisons may vary according to the direction of comparison (upward vs. downward) as well as the internal process that triggered by those comparisons (assimilation vs. contrast). The current study aims to examine the mothers' emotions they felt after social comparisons they make with other mothers on the SNSs, and search out the usefulness of using the typology of social comparison-based emotions in examining those emotions. For this purpose, an online survey was conducted on 42 mothers between the ages of 20 and 48, who have been a member of SNSs for at least six months. Mothers responses to two open-ended questions; one is about the emotions they feel after upward comparisons, and the other is about the ones that they felt after downward comparison they made with other mothers on SNSs, were examined through thematic analyses. The results pointed out that the emotion classification offered in Smith’s theory might be useful in examining the social comparisons on SNSs made by mothers, with the addition of some new categories. Specifically, it was found that some mothers feel doubts about the credibility of information in other mothers’ posts, and some others denied they are emotionally influenced by social comparisons. Another interesting finding was that mothers reported to feel assimilative and contrastive emotions simultaneously.

Social networking sites (SNSs) are worldwide platforms that allow mothers to make social comparisons as well as to get social support and information about motherhood (Bartholomew, Schoppe-Sullivan, Glassman, Kamp Dush, & Sullivan, 2012). As social comparisons have a potential to evoke a wide range of positive and negative emotions that may produce some significant consequences for individuals’ psychological well-being and depression (Appel, Crusius, & Gerlach, 2015; Feinstein et al., 2013; Park & Baek, 2018), the emotions mothers feel after making social comparisons with other mothers on their SNSs is worthy of being studied.

## Theoretical Background

SNSs facilitate social comparisons among people (Haferkamp & Krämer, 2011). The direction of social comparisons on the Internet is usually upward since people often tend to present their idealized self, rather than their true self, during their communication through the internet channels (Walther, 1996). Supportively, it has been observed that people tend to post the pictures and videos in which they are the most beautiful/most successful/the happiest in their digital profiles (Chou & Edge, 2012). Therefore, it came as no surprise that in most of the studies about social comparisons on SNSs, the main focus has been upward comparison and its negative consequences, such as depression (Feinstein et al., 2013; Lee, 2014; Locatelli, Kluwe, & Bryant, 2012).

In various studies, women reported more adverse outcomes than men when evaluated after social comparisons (Chua & Chang, 2016; Fox & Vendemina, 2016; Nesi & Prinstein, 2015). There are several domains that women may compare themselves with other women on their SNSs: Their careers, domestic skills, and femininity, etc. Along with these ongoing social analyses, when a woman gives birth to a child, motherhood becomes a new domain for social comparison. During their pregnancy, women share the narratives of various physiological and psychological changes that they experience with others, thus the progress in motherhood role adaption may be a topic of social comparison for those to be expected mothers (Phillips & Broderick, 2014). After giving birth, mothers may compare their maternal competence (Coyne, McDaniel, & Stockdale, 2017).

Mothers make social comparisons with non-mothers as well. Being a mother may lead mothers to feel superior to non-mothers because of a widespread cultural belief that motherhood is essential for women’s psychological fulﬁllment, and a good marriage and family are considered strongly desirable for all women (Hoffnung, 2004). However, being a mother may likewise lead mothers to feel inferior to non-mothers in some comparison domains. Some consequences of pregnancy, such as weight gain, bodily deformations, postpartum depression, and maternity leave may lead new moms to perceive themselves as inferior to non-mothers in terms of comparison areas like beauty, career achievements, and happiness. Given that people tend to make social comparisons with similar others, mothers may prefer not to use non-mothers as targets in their social comparisons regarding career and beauty. Instead, they may make comparisons with the other mothers in terms of their competence in coping with the potential threats to their beauty and career achievements that emerge after being a mom. There are emotional consequences of all those social comparisons that mothers may make with other moms. Those emotions are worth to be studied in detail because they may have implications for their wellbeing. For detailed analyses of mothers’ social comparison experiences, the theory on social comparison-based emotions may be considered as a useful framework.

## Theory on Social Comparison-Based Emotions

The earliest theory for explaining the social comparison process in interpersonal relationships is Festinger's (1954) social comparison theory. According to Festinger (1954), people frequently compare themselves with others in their everyday lives in various ways. Those comparisons might be either upward or downward. One of two alternative internal processes can be triggered as a result of those comparisons: Assimilation vs. contrast (Tesser, 1991). When the assimilation process follows upward comparisons (UCs), the comparers consider the comparison targets as models for themselves. They become hopeful that in the future, they might be like those target people. On the contrary, when a contrast process follows UCs, the comparers focus on the differences between them and the target people. As a result, they feel negative emotions (e.g., jealousy, helplessness). Downward comparisons (DCs) may also lead to processes of assimilation or contrast. Assimilation with the DC targets can lead to negative thoughts and feelings (e.g., “Will I be sharing a similar bad destiny with them?!”). However, the contrast process may lead to positive thoughts and emotions (“Thankfully, I am not like them”). Whether a social comparison evokes assimilation or contrast process may depend on how close the comparer feels towards to the comparison target: If the comparison target is a close person, social comparisons are more likely to end up with assimilation, but if the comparer is not psychologically close to the target, then, a contrast process is more likely.

Based on all this information, Smith (2000) mapped out many possible emotions that might arise from social comparisons. In his typology, he differentiated four emotion categories based on the direction of the social comparison (up or down) and the process triggered (assimilation or contrast). He claimed that predicting which emotions may come down after social comparisons may depend on various factors, such as how likable or dislikable the comparison target, how significant the self-other discrepancies, and to what extent those discrepancies are changeable, or to what extent they are a result of relatively even actions, etc. The emotions that fall into each of the four categories in his typology was given below:

**1.** Upward contrastive emotions (UCEs): Depressive feelings and shame, resentment, and envy.

**2.** Upward assimilative emotions (UAEs): Inspiration, optimism, and admiration.

**3.** Downward contrastive emotions (DCEs): Contempt/scorn, schadenfreude, and pride.

**4.** Downward assimilative emotions (DAEs): Fear, pity, and sympathy.

In the current study, those emotions of Smith’s (2000) typology are accepted to be universal, but we also acknowledge that there can be some variations in emotions within each category depending on culture or language. For example, although Smith defined jealousy as an UCE, it should be noted that there are two different forms of jealousy - benign and malignant- in some languages such as German, Dutch, and Russian (van de Ven, Zeelenberg, & Pieters, 2009). The former one seems to better suit into the category of UAE while the latter one is a UCE. Another example is the feeling of fear-of-evil-eye. In Smith's (2000) system, fear is categorized as a DAE. Smith has not defined fear-of-evil-eye as a distinct emotion. However, in cultures where evil-eye belief is powerful, social comparisons also may produce this belief-specific emotion (Sabuncuoğlu-İnanç & Saatçioğlu, 2017). Fear-of receiving the evil-eye is very common in Asian, African and Mediterranean regions of the world. The evil-eye known as “nazar” is also a popular folk belief in Turkey. It is a belief that when one envies others, a failure is likely to be experienced by the envied (e.g., if one grudges another person’s possessions like wealth or beauty, something terrible might happen to that envied person which cause harm). The fear-of-evil-eye is an emotional consequence of this superstition (Shoeck, 1981). This fear leads people to take some preventive measures: Well-to-do people try not to show off, not to talk loudly about their possessing to others to protect themselves from possible damages or bad luck that may stem from being envied. The fear-of-evil-eye does not fit well into any of the categories within the Smith’s (2000) system, because it is not an emotion that people feel by focusing directly on superiority/inferiority of either oneself or the other. Instead, fear-of-evil-eye emerges from a metacognitive focus (e.g., “What would she feel if she judged that I am superior to her? Would she envy me?”). In sum, we argue that utilizing Smith's four emotional categories could be very helpful to understand emotional variation in different social comparison contexts, if researchers pay also attention to language and context-specific selection of emotions.

In the current study, we aimed to explore what kind of inner processes and emotions that mothers experience following their social comparisons with the other mothers on their SNSs. We aimed to examine the emotional consequences of mothers’ social comparisons with other mothers not only in terms of motherhood but in any areas that they consider to be relevant. We asked the mother to tell us their emotions after upward and downward social comparisons with other mothers on SNSs, and we examined the emergent themes in their responses. We tested the usefulness of Smith’s (2000) social-comparison-based emotion categories in this examination. To the knowledge of the authors, there is only one study that tests Smith’s social comparison-based emotions in the online context of SNSs (Park & Baek, 2018). This study is one of a kind that social comparison-based emotions are examined in the motherhood context. Our main aim was to evaluate whether the emergent themes in the current study would map with the four categories in Smith’s (2000) classification. We also aimed to observe whether any additional and culture-specific themes or/and emotions would emerge. A culture-specific hypothesis also asserted that the fear-of-evil-eye would also emerge as one of the constructs under the downward contrastive emotional theme for Turkish mothers.

## Method

### Participants

Data were collected from 42 Turkish respondents. The age range was from 20 to 48 years. The *M*_age_ = 36.36 (*SD* = 6.91). Respondents reported that they have either one or two children. Birth dates of children varied between 1992 and 2017.

Most respondents reported having higher education (54.8%), having a job (69%), living in a metropolis (50%), placing themselves on the 4th step (42.9%) of a 7-step ladder representing socioeconomic status (SES) from 1 (the lowest SES) to 7 (the highest SES).

About the usage of social networking sites (SNSs) since the last six months, most respondents (over 50%) reported being members of at least two sites. The majority were members of Facebook (95.2%) and/or Instagram (71.4%). Most of the users (37.5%) stated that the number of their SNS friends were between the range of 201-400. Respondents were asked the duration of daily time they spent in each of their various SNSs in the last week. The favorite response was 10-30 minutes for both Facebook use (32.5%) and Instagram use (26.7%). Finally, most respondents reported that the last time since that they shared a message or post something on SNSs was a few days ago (28.6%); the last time since that they followed others’ posts on SNSs was that day (71.4%). More detailed information about the responses to the demographic questions and the questions about SNS use was given in Table 1.

**Table 1:** Demographic Characteristics of the Sample and the Information About Participants’ Usage of Social Network Sites (N = 42)

Variable	Percentage
Place of residence	9.5% live in a rural area; 40.5% live in a city; 50% live in a metropolis
Socio-economic level (the 1st step reflects the lowest socio-economic level and the 7th step is the highest)	2.4% are on the second step; 9.5% are on the third step; 42.9% are on the fourth step; 33.3% are on the fifth step; 9.5% are on the sixth step; 2.4% are on the seventh step
Education	16.6% have secondary education degree; 54.8% have higher education; 28.6% have graduate degree
Employment	79% have a job; 12% are unemployed but looking for a job; 19% are unemployed and not looking for a job
Profession	23.8% are teachers; 16.8% are health-care professionals; 9.6% are academics; 4.8% are accountants; 30.7% are other professions (e.g. banker, computer operator, child development specialist, economist, housewife, chemical technician, market owner or employee, student, procurement manager, self-employed, insurance agent, political scientist, chairman of the board); 14.3% do not have any profession
The usage of SNSs in the last six months	23.8% have one SNS; 50% have two SNSs; 21.4% have three SNSs; 4.8% have four SNSs; 95.2% use Facebook; 71.4% use Instagram; 7% use other SNSs (e.g. Twitter, WhatsApp, Snapchat, Pinterest, and Swarm)
Number of friends on Facebook	30% have less than 200 friends; 37.5% have a range between 201-400 friends; 32.5% have 401 or more friends
Duration of daily time spent on Facebook	15% spend less than 10 minutes; 32.5% spend 10-30 minutes; 17.5% spend between 31-60 minutes; 20% spend between 1-2 hours; 5% spend 2-3 hours; 10% spend more than 3 hours
Duration of daily time spent on Instagram	10% spend less than 10 minutes; 26.7% spend 10-30 minutes; 16.7% spend 31-60 minutes; 16.7% spend 1-2 hours; 10% spend 2-3 hours; 20% spend more than 3 hours
Time of the last post on SNSs	%26.2 shared in that day; 28.6% shared a few days ago; 19% shared a few weeks ago; 11.9% shared a month ago; 14.3% shared more than a month ago
The last time others’ posts have been watched/read on SNSs	71.4% watched/read in that day; 26.2% watched/read a few days ago, and 2.4% watched/read a few weeks ago

*Note.* SNSs = Social network sites.

### Procedure

We posted an open call to our SNSs and gave a link to an electronic survey, using the service provided by “surveey.com”. Volunteers, who provided the preconditions, filled out the questionnaire. There were two preconditions: 1) to be a user of at least one SNS (Facebook, Instagram and so on) for at least the last six months, and 2) to be married and have at least one child. We also requested the mothers to post the call to participate to our study on their SNSs to reach some other mothers who would like to volunteer in our research. Respondents were first presented informed consent, and then, they filled a three-part questionnaire, involving the demographic information form, the SNS usage information form, and finally, questions about emotions felt after social comparisons in SNSs. At the end of the data collection process, respondents have been thanked and debriefed.

### Measures and Instruments

#### Demographic Information

The form included questions about mother’s age, education level, occupation, household income, place of living, number of children, and age/s of their children.

#### SNS Usage Information

Respondents were asked about having memberships of which SNSs (Facebook, Instagram, etc.), the size of their network (number of friends for each SNSs) and how often they use these networks (frequency of viewing and sharing).

#### Questions About the Emotions Felt After Social Comparisons in SNSs

Two open-ended questions were asked about the emotions. One question was about the emotions respondents feel after UCs; and the other one was about the emotions after DCs. The question for the UCs is given below:

“Sometimes, mothers follow what other mothers share in the SNSs and make social comparisons with those mothers. In the current study, we would like you to imagine yourself while browsing one of your SNSs (Facebook, Instagram, etc.) and following the posts of other mothers. Imagine that at the end of your browsing, you come up with a judgment that those mothers are healthier and more beautiful than you are, that they have more money and time to do what they want, that they have better parenthood skills than you do. Some emotions might accompany those thoughts. Imagining that the browsing mother was you, which emotions do you think you would feel? Please write freely about the emotions you would feel, how intense they would be, and which situations would lead you to feel each of those emotions.”

The question for the DCs was similarly worded: First, respondents were asked to imagine themselves while making a downward comparison on the SNSs, then, they were asked to report us about what would they feel, how intense those emotions would be, and which situations would lead them to have those emotions.

### Data Analysis

A multi-stage thematic analysis process suggested by Braun and Clarke (2006) was conducted. The analyses were performed separately on the responses to the questions about emotions feel after UCs, and DCs.

The first step for the analysis was to read the text to gain familiarity. Next, codes were generated, and then, they were combined into themes, next, those themes were reviewed, defined and named. Finally, all the themes were summarized and interpreted. Two judges coded the data independently and then a meeting was conducted to overcome any inconsistencies in their coding. During the coding, a balance of deduction (deriving themes from the theoretical framework) and induction (emerging of themes from respondents' responses) was sought. More specifically, whether Smith’s (2000) concepts of assimilative and contrastive emotions were mapped effectively to the current data set was examined first, and then, codes that did not fit into assimilation-contrast framework were reviewed to see if they would form new themes.

## Results

Thematic analyses on the responses to the question about emotions felt after UCs showed that, a priori themes – UAEs and UCEs- were related to the data. Some new themes for UCs also emerged, and named as “UC denial and doubt”, “and “UC emotions felt in the name of others”. The results of thematic analyses on the responses to the question about emotions felt after DCs also showed that a priori themes – DAEs and DCEs- fit into the data as well. Again, some additional themes for DCs came up. These were named as “DC denial and doubt” and “DC emotions felt in the name of others.” Each of a total of nine themes is summarized and interpreted below.

### Emotions Felt After Upward Comparisons

#### UC Contrastive Emotions Theme

Some respondents reported feeling negative emotions (envy, resentment, sadness, etc.) after UCs. However, mothers appeared cautious while disclosing experiencing negative emotions: They have specified negative emotions only on some certain comparison topics or only towards limited comparison targets and to small extent. Examples of utterances about negative emotions after upward contrasts; “I would envy the trips that others did”, “I would feel a little envious only when the others are someone whom I don’t like”, “I would not feel emotions of sadness and anger very intensely”. On the other end of the pendulum, some respondents have stated that they would not experience these emotions in the utterances like “I guess I wouldn’t feel envy”, “This situation wouldn't make me feel sad or bothered”. Others stated that they would substitute them with some other emotions, thoughts or even actions such as “Rather than feeling envy, I would think that everyone has different standards, and I mind my own business”, “Of course I would feel sorry, but I would also do something about it”. The fact that respondents mentioned the emotions of envy and resentment, even if it is for indicating that they would not experience these emotions or they would substitute them with other emotions, actually signals that these two are among the basic social comparison emotions. Among the UCEs, sadness and dissatisfaction came up as the two emotions that respondents seem more at ease to disclose that they experienced (e.g., “I would be sad in such a situation”, “I would feel very dissatisfied”, “I would feel unhappy”, “I would feel that I was inadequate, -especially as a mother- and I would feel pity for myself”). Some respondents also mentioned the feeling of anger. Rather than as an emotion triggered by social comparisons, anger appeared as an emotion directed to the blogger mothers that blog with commercial purposes. –Mothers’ don’t seem to exert anger as a result of asking oneself “why is she better than me” or “why am I not like her?”, instead anger as an emotion seems to be triggered by thinking that the blogger mothers lie to their followers. For instance,

Respondent #42: “Comparing one’s own family with such people –especially in terms of financial means- has become one of the reasons for conflict in the family. Some mothers ridiculously become bloggers, and they take their kids to certain places for eating and drinking or for playing. It is weary. They try to maintain their social media image as good mothers in this way. They sometimes make a contract with business owners to advertise their places for receiving free eating, drinking, and shopping. What they do may cause financial conflicts between the spouses. What people understand from “socializing” is that going somewhere, and enabling notifications of these places and making an Instagram story. They put background music, and tell at which cafe that they have been and what they have eaten. They pretend that they have been in that cafe for their kids. They label the shopping website they bought their clothes and sunglasses -they make sure to post at least two photos with sunglasses. They end their story with a quote like “it was a tiring but nice day.”

One of the respondents mentioned that she would blame herself after UCs on SNSs. Although the feeling of inadequacy is not one of the UCEs defined by Smith, it is reasonable to consider it as applying to that category.

Respondent #17: “Perhaps I would blame myself for anything that I did not and could not have. I might continue reflecting on this negative energy to my significant others.”

#### UC Assimilative Emotions Theme

UCs may lead to feelings of optimism, admiration, and inspiration if they trigger the assimilation process (Smith, 2000). However, none of the respondents in this study mentioned these three emotions. Nevertheless, there were some expressions in the respondents’ statements indicating that they have experienced an assimilation process after UCs. Some respondents have reported that they would take the comparison target as a model. The research question was just about their emotions, and it was not our scope to cover their behavioral intentions. However, respondents preferred to declare what they would do (taking the target person as a model) rather than what they would feel in the assimilation process.

Another emotion that can be acknowledged as the UAE was the feeling of appreciation. However, respondents clearly stated that they would appreciate a comparison target only if the positive features that she informs on SNSs were honest. From the expressions of those respondents, it was clear that they were doubtful about the reliability of personal information entered into SNSs.

Respondent #20: “As much as I can, I try to follow the mothers whose kids are the same age as mine. If they inform that they accomplished some progress in experiences with their kids, then I try to do whatever they did. In other words, I take them as a model.”

Respondent #21: “I would take them as a model and bring something new into my life accordingly.”

Respondent #36: “I would feel a little bit inadequate, and I would wish to behave like them.”

Respondent #29: “I model their positive aspects of their life experiences if they are people whom I love and value, and I try to take them as models. If the people whom I do not like are outstripping me, then I feel envy.”

Respondent #34: “I feel sorry, especially if they are superior in maternal aspects. But if those people are my friends and if I believe that they are genuinely sincere and realistic, then I take them as models, or begin to search to become a better mother.”

Respondent #4: “Believe me, I congratulate a successful person by the heart. If she deserves what she gained, may God clear her path.”

Respondent #41: “It would be nice if she has managed her life well. But is it really so? I would appreciate her if she is honest about performing well.”

Interestingly, following the expressions above, a conceptual controversy seemed to emerge. Some of the respondents referred “taking as a model” and the UCEs in the same phrase: For instance, the Respondent #36 used the expressions of “feeling inadequate” and “doing something as they (the UC targets) do” together; Respondent #29 used the utterances of “feelings of envy” and “taking as model” together; and Respondent #34 used the expressions “sad and inadequate” and “taking as models” together. From these expressions, it is evident that after making a social comparison, both assimilation and contrast processes may simultaneously evoke within the same person.

#### UC Denial and Doubt Theme

This category mainly covers responses reflecting either of the reactions below:

**1.** Denial of that one does social comparisons in SNSs or denial of that one is emotionally affected by social comparisons:

Respondent #37: “I do not feel anything. I think they are luckier than just talented. But it does not disturb me.”

Respondent #13: “I do not care about what others have or how they look. Every human being is different in his or her living conditions and life philosophy! I AM ME, and THEY ARE THEM! I do not feel anything when I read the profiles of other people!”

**2.** Disbelieving that the social comparison target is superior to oneself:

Respondent #2: “I would not be very impressed because, in social media, everyone is in a position to show himself as perfect, but it is fake.”

Respondent #30: “I would not care much about what I see in social networks. I do not think they are sincere and realistic. I think it carries the character of showing off."

**3.** Self-affirmation efforts to buffer against social comparison threats:

Respondent #3: “Other’s thoughts and feelings about me are unimportant for me. I always act with my own free will.”

Respondent #4: “... I do not weigh my worth according to someone else’s opinions. I know that I'm valuable.”

Respondent #27: “I have what my God predestinated. I wish Allah will bless me with a better life than what I have now, but even if I don't have a better life than now, it is not a big deal. I am a decent and nice mother. When I see a smile on my child's face, this makes me feel good enough. A better financial situation might make us live a more luxurious life, but if we cannot afford that, it is not a problem. Having my husband and my daughter with me is enough Alhamdulillah.”

#### UC Emotions Felt in the Name of Others Theme

Some respondents have expressed what they would feel on behalf of the target of comparison, not on behalf of themselves, after upward comparisons. The emotions in this category were all positive emotions. However, one of the respondents (Respondent #5) stated that feeling either positive or negative on behalf of another mother could also be related to her own mood at the moment.

Respondent #8: “Their happiness makes me happy too.”

Respondent #5: “If I were happy, the good things about others would make me happier, while the very same things would be meaningless for me if I were sad. I feel positive feelings very intensely. I try to suppress negative emotions.”

Respondent #14: “... I welcome other people’s good news; I am pleased that I hear them. I “like” them.”

### Emotions Felt After DCs

#### DC Contrastive Emotions Theme

Emotions reported by the majority of the mothers in the downward contrastive category are on behalf of themselves and positive.

Respondent #1: “How lucky I am.”

Respondent #5: “A feeling of strong gratitude. A mildly strong feeling of fear from the evil-eye" (this expression has been expressed together with the statement of "a strong desire to help”).

Respondent #36: “I would feel pleased, and I would be proud to be such a mother.”

Respondent #35: “In such a case, I would be happy on behalf of myself, but at the same time I would feel sorry for behalf of others. I wish everyone to be able to raise their children in the best possible conditions.”

Among the above examples, the emotions specified by Respondent #5 were particularly striking. She expressed that she would feel all the 1) gratitude, 2) fear-of-evil-eye, and 3) a desire to help another person. To us, it was interesting that Respondent #5 was the only respondent that mentioned fear-of-evil-eye. Among her phrases, the feeling of gratitude was classified as a contrastive emotion, while the desire to help others was classified as an assimilative emotion. Gratitude was regarded as contrastive emotion because of its being associated with the thought of “I am happy that I am not like her.” However, to feel gratitude, it is also necessary for one to think as “I might be in her shoes.” In this sense, gratitude can also be regarded as an assimilative emotion. It should also be noted that the expression of gratitude is an indicator that the respondent makes an external attribution for the discrepancy between the self and the inferior other. It should also be noted that by reporting experiencing both (gratitude and desire to help), she provided us with an example of how assimilative and contrastive emotions can be experienced simultaneously. Although the desire to help is not a pure feeling but rather “a behavioral intent”, it is on the midpoint of being an emotion and being a behavior. In this sense, it is similar to the intent of "taking someone as a model" which is expressed after the upward-contrast process.

#### DC Assimilative Emotions Theme

This category involves other-focused emotions such as anger for the injustice that others experience, and a desire to help others. It is noteworthy that “a desire to help others” appeared as a part of both downward-contrastive and downward assimilative emotions themes.

Respondent #8: “Their good news makes me happy. I feel so sorry when I see people post about their sorrows.”

Respondent #12: “I would feel sorry when I see some situations which I consider as unfair. I would think about how I can help mothers or how I can make a difference for them. I would do anything for that aim.”

Respondent #21: “I am sorry for the people who do not have the same opportunities as mine, and I try to support those people if I have any means to do it.”

Respondent #37: “I would think that I'm luckier than others. I would not be happy to be in a better position than them. I think that is a shame that not everyone has adequate standard of living.”

Respondent #41: “I feel sorry for the unluckiness and passivism. I feel anger at the injustices that our education system has done to the woman.”

#### DC Denial and Doubts Theme

Some of the respondents have denied that they were doing DCs, or they have denied that the emotions that they experienced after DCs. Some said that they do not trust the accuracy of the information that people reveal about themselves in SNSs. One of the respondents (Respondent #34) has labeled "playing house on social media" to other mothers' postings of unrealistic messages in SNSs.

Respondent #7: “I do not compare myself to anyone.”

Respondent #13: “I would not feel anything different because I do not care about social media so much to let it capture my soul!”

Respondent #30: “In general, I would not feel that I was luckier. Again, I think people do not tell the truth in social media. I do not overreact to such mothers in social media. I do not believe that social media reflects reality with one hundred percent truth.”

Respondent #34: “... I do not believe in mothers’ playing house on social media.”

#### DC Emotions Felt on the Name of Others Theme

Some respondents have pointed out not only the emotions they would feel for their behalf but also the ones they would feel for someone else's behalf after a downward comparison. One of the respondents (Respondent #34) stated that she would be sorry for both the target person and her children. Another participant reported that she would feel happy on behalf of her own children (Respondent #16). Another unique answer was Respondent #35’s. In the first part of her response, she stated that she would feel a UCE (“In such a case I would be happy on behalf of myself” …), but in the second half of her response, she stated that she would feel a DCE (“…but at the same time I would feel sorry for behalf of others.”). With this answer, the Respondent #35 provides an example of that a UAE, and a DCE may be felt simultaneously or, at least consecutively. It seems that the respondent first does a UC. Next, she takes the perspective of the other mother and relives the situation as she does. Therefore, the comparison might have changed the direction -UC has turned into a DC, and it created sadness. In other words, a single comparison led to the feelings of happiness and sadness in succession.

Respondent #16: “Happiness is the happiness of my children.”

Respondent #34: “I feel sorry for these people. I am sorry for the child.”

Respondent #35: “In such a case, I would be happy on behalf of myself, but at the same time I would feel sorry for behalf of others. I wish everyone to be able to raise their children in the best possible conditions.”

## Discussion

In the current study, the aim was examining mothers’ emotions which they feel after making social comparisons on SNSs with other mothers, and searching out to what extent Smith’s (2000) social-comparison-based emotion categories were useful for exploring those emotions. It was seen that a wide range of emotions were elicited after mothers’ social comparisons on SNSs with other mothers, and many of those emotions fitted well under the themes of assimilative and contrastive emotions, as proposed by Smith (2000).

The current study also has provided some examples of that the assimilation and contrast processes might be both triggered -either simultaneously or successively- after a particular social comparison. This result showed a deviation from Smith’s (2000) conceptualization of social comparison-based emotions: According to him, assimilation and contrast were two alternative processes, and he has never mentioned that a social comparison may evoke both. Another interesting finding was that social comparisons of mothers, trigger not only some emotions, but also some behavioral intentions. More specifically, UCs seem to be triggered the behavioral intention of taking the superior other as a model; and DCs triggered willingness to help others. These findings provided a support to the idea that behavioral intentions and emotions are tightly coupled domains, and should be evaluated together in social comparison literature.

The findings also revealed supportive evidence for the existence of four categories (UCEs, UAEs, DCEs, and DAEs) described by Smith's (2000) emotion-based social comparison typology. However, emotions included in each category in the current study have not strictly matched with the ones offered by Smith (2000). Some of the emotions listed under those four categories in Smith’s typology have never come up in the current study. For instance, optimism, admiration, and inspiration were suggested as upward assimilative emotions in theory, which our respondents have never mentioned. Instead of reporting what they would feel, respondents in general preferred to write what they would do after UCs, and stated that they would take the superior comparison target as a model to themselves. It may because that our question format was inclusive, and probably, mislead that the question was about actions rather than about emotions. Also, no respondents have mentioned that they would experience two of basic theory driven DCEs (contempt and schadenfreude), and one of the basic DAEs (pity). Respondents may have opted not to report negative emotions because they are undesirable emotions. Contempt and schadenfreude are socially undesirable emotions as they indicate devaluing of others and produce antisocial behaviors (Weiner, 2005). However, it is debatable whether pity is a desirable or undesirable emotion. At one hand, pity can be considered as a positive and prosocial emotion that is shown under the condition that the others are incompetent and their incompetence is attributed to uncontrollable deficits (Weiner, 2005). On the other hand, it can be considered as an ambivalent emotion from the point of view of the target person: This emotion may be pleasant for the target person because it shows that the others do not treat them with indifference, but it may also hurtful because it reminds them their own weaknesses and incompetency (Ben-Ze'ev, 1993). Because of this ambivalent characteristic of pitying, respondents might prefer not to declare that they would feel pity for the inferior others. Instead of pitying, “a desire to help others” has emerged as mothers’ outstanding motivation in the current study. “A desire to help others” may be considered as a concept broader than feeling pity, and also it is more polite and less heartbreaking. It is no surprise that our respondents refrain from being rude and heartbreaking: According to gender stereotypes women are kind, and sensitive to others’ needs; and according to motherhood myths, mothers are “more women” than non-mothers (Sever, 2015). As our questions made their maternal identity salient, respondents probably refrained from expressing heart-breaking negative emotions which would not fit into motherhood identity, and they tried to show the positive emotions and socially desirable behavioral intentions that suits well with a divine mother image.

Another interesting finding is that anger appeared as a DAE in the current study, although it has been described as a UCE in Smith’s (2000) theory. In our study, mothers’ anger seemed to be targeted the injustices suffered by others, rather than the superior others. It appears that some mothers who are exposed to DC targets on the SNSs look at the world from the eyes of those DC targets, and focus on the external conditions that make them suffer. The emotional and motivational predictors and consequences of mothers' sensitivity evoked by others’ vulnerabilities on SNSs need a further interest in future studies.

The reply by the Respondent #5 to the UC question is worth to be discussed by its own. This response includes an explanation about the mood depending nature of positive or negative emotions after UCs. By this reply, the respondent brought up the issue of the moderating role of mood in the links of social media information with elicited emotions. According to her, after expressing an UC, assimilative emotions might be evoked if the person is in a good mood. However, contrastive emotions might evoke if the person is in a negative mood. Previously, it was shown that when people are given a chance to select which social comparison target to be exposed to (UC vs. DC) in a SNS browsing task, their decisions is affected by their mood. When they are in an aversive mood, they are less likely to select UC targets, and more likely to select DC targets (Johnson & Knobloch-Westerwick, 2014). Experimental design studies might help to elaborate on the question of whether the individuals’ assimilative vs. contrastive type of emotional reaction depends on their comparison specific mood.

Our results also showed that some respondents chose not to believe in the accuracy of information provided by others in SNSs, and they claim that it might be fake or exaggerated (denial strategy). This strategy has been used both in the UCs and DCs. In the literature, there are predictions and some supportive research findings regarding how individuals' self-descriptions on SNSs reflect the ideals, not the facts (Walther, 1996). Because of the abundance of false information and fraud on SNSs, deciding whether information on SNSs is true or false is a legitimate issue for the users of those platforms (Walther & Parks, 2002). The respondent mothers’ reported doubts and denials in the current study might be reflecting their awareness of misleading behaviors on SNSs.

According to the literature, individuals may use self-affirmation as an alternative strategy when they face with self-threatening information (Sherman & Cohen, 2006). In the current study, we see the example of that some respondents use the self-affirmation strategy after making upward comparisons. More specifically, some mothers who make social comparisons on SNSs reported that they were able to move negative feelings and behaviors triggered by such comparisons away through reflecting on their prominent values in their lives unconnected to the domain of comparison. While the current study provided some examples of how SNSs create a need for self-affirmation; in the literature, there is supportive evidence showing that users also use those sites to gratify their need for self-affirmation. Previously, Toma and Hancock (2013) demonstrated that individuals use their social media profiles to self-affirm when they need to repair their self-worth.

After both UCs and DCs, some respondents have expressed their emotions not only on their behalf but also on behalf of the comparison targets. Happiness reported as the most frequent emotion to be felt in the name of others, after the upward comparisons. The sadness appeared as the most often cited emotion to be felt in the name of others after downward comparisons. In Smith’s (2000) theory, social comparison emotions are assessed in two dimensions: Valence (positive vs. negative) and focus of comparison (self-vs. other). "Emotions felt on behalf of others" is not a category defined in Smith’s theory. To avoid any confusion, there must be an explanation about how “emotions felt on behalf of others” is conceptually different than “other-focused emotions." The former one involves the probable replies to the question of “What do I feel when I put myself into her place?” whereas the latter one involves response to the question of “What do I feel for her?”. In the current study, the fact that respondents frequently mentioned which emotions they would feel on behalf of others may be considered as an indicator of the relational self.

It should be noted that data were collected from Turkish mothers, and Turkish culture is a culture of relatedness that emphasizes emotional interdependency for its people (Kagitcibasi, 1996). Thus, in the current study, it is no surprise that many respondents have inclined to evaluate a situation not only from their own perspective but also from the viewpoint of target person. Although they were specifically asked to report their own emotions after social comparisons, they have reported more on what they would feel on behalf of target person.

In the current study, the fear-of-evil-eye emerges as one of the constructs under the downward contrastive emotions theme, as hypothesized. This result supports the findings of an earlier study conducted in Turkey demonstrating how people’s fear-of-evil-eye and their efforts for getting protection against the evil-eye are transmitted from offline to online life through the examination of Instagram hashtags (Sabuncuoğlu-İnanç & Saatçioğlu, 2017).

### Limitations

There are several methodological limitations of the current study. First, participants were asked a very general question about what they feel after social comparisons with other mothers on SNSs. If separate questions for each comparison domain, such as health, wealth, and attractiveness, had been asked, participants might give different responses to each. In the current study, it was assumed that participants would consider the question by depending on how important each social comparison domain for them and answering for the “one” that is most important to them. In further studies, we recommend researchers asking separate questions for each of several comparison domains, and compare the emotional consequences of comparisons in each domain with each other. The second limitation is that every participant was given the question about UC and DC in the same order (UC first, and DC later). As some literature suggested that making UCs increases negative mood, we tried to protect participants from leaving the session with a negative mood by refraining from asking the UC-question at the end of the session. However, this could have resulted in order effects. In further studies, researchers may consider doing counterbalancing. Lastly, the broad age range of children of the sampled mothers in the current study might be a point of criticism. In the future, it might be a better idea to do studies in which the focus was mothers with infants and young children because those mothers are more likely to be vulnerable to social comparison.

### Conclusions

Many of today's mothers integrate themselves into digital life and use SNSs for making social comparisons. Those emotions have a potential to evoke various emotions. The current study has shown that Smith’s (2000) typology for social comparison-based emotions -with the addition of some other emotion categories- provides a well-depicted framework for studying mothers’ emotional reactions that are evoked by others in digital networks. As offline contexts seem to give more space to express feelings of all kind, future research utilizing different methodologies should continue to examine and compare social comparison-based emotions in both online and offline contexts.
